# A Novel Method for Simultaneous Production of Two Ribosome-Inactivating Proteins, α-MMC and MAP30, from *Momordica charantia L*

**DOI:** 10.1371/journal.pone.0101998

**Published:** 2014-07-08

**Authors:** Yao Meng, Sen Lin, Shuangfeng Liu, Xiang Fan, Gangrui Li, Yanfa Meng

**Affiliations:** 1 School of Medical Laboratory Science, Chengdu Medical College, Chengdu, China; 2 Department of Histology and Embryology and Neurobiology, Development and Regeneration Key Laboratory of Sichuan Province, Chengdu Medical College, Chengdu, China; 3 Key Laboratory of Bio-resources and Eco-environment Ministry of Education/Animal Disease Prevention and Food Safety Key Laboratory of Sichuan Province, College of Life Science, Sichuan University, Chengdu, China; University of Helsinki, Finland

## Abstract

Alpha-momorcharin (α-MMC) and momordica anti-HIV protein (MAP30) from *Momordica charantia L*. have been confirmed to possess anti-tumor and anti-virus activities. Traditional purification methods of these two ribosome-inactivating proteins (RIPs) were separate which was time consuming and cost effective as well as low efficient. In order to obtain sufficient samples for researches, a strategy combining ion-exchange and gel filtration chromatography was developed and optimized in this study. Using this novel purification method, averagely 1162 mg of α-MMC and 535 mg of MAP30 were obtained from 400 g of *Momordica charantia L* seeds. The homogeneities of them were assessed by electrophoresis analysis. Determination of molecular weights of α-MMC and MAP30 were 28.585 kDa and 29.094 kDa by MALDI-TOF/TOF and pI were 9.02 and 9.12, respectively. The single glycoproteins were identified by Periodate-Schiff's base (PAS) and the saccharide content was tested to be 1.25% and 1.1% by anthrone-sulfuric acid method. Biological activities were evidenced by their ability to inhibit proliferation of lung adenocarcinoma A549 cell and to convert supercoiled plasmid pUC18 into relaxed forms. Finally, we also found that both two RIPs exhibited no superoxide dismutase (SOD) activity.

## Introduction

Ribosome-inactivating proteins (RIPs) were firstly discovered in plant following the research towards to ricin [1]. In recent years, researchers commonly categorized RIPs into three types, referred to as type I, type II and type III [2]. *Momordica charantia L*. (MC), a *Momordica Linn*. genus of the family *Cucurbitaceae*, also known as bitter melon, is served as a traditional medicine plant by Chinese from ancient to modern times [3], [4]. Further research confirmed that it belongs to type I RIPs which are single-chain proteins with a molecular weight of approximately 30 kDa and is commonly localized in plant leaves, seeds or roots. Alpha-momorcharin (α-MMC) and momordica anti-HIV protein (MAP30), two major components in MC, were found to be potent inhibitors of protein synthesis due to their ribosome-specific *N*-glycosidase activity [5]. Accordingly, α-MMC and MAP30 have been shown to exhibit antitumor activity *in vitro* and *in vivo*, including lung, colon, liver, epidermis, breast cancer and melanoma cells [6]–[8]. Meanwhile, both of them also have broad-spectrum antiviral activities against different viruses, such as herpes simplex virus-1 (HSV-1) [9], hepatitis B virus (HBV) [10], and acquired human immunodeficiency virus type-1 (HIV-1) [11], [12]. In the light of these findings, high-quality samples are needed for further researches, especially in our previous work focused on PEGylation which can lead to significant reductions of immunogenicity [13]–[15]. Herein, we develop a simple, convenient and repeatable purification method for the two RIPs, particularly during which they can be produced simultaneously in a one-step purification process. In the manuscript, this novel method employed SP-Sepharose FF, Sephacryl S-100HR and Macro-Cap-SP as chromatography media. Further studies we determined their structural characteristics and biological activities. To our knowledge, this was the first study pertaining to a large-scale preparation of both α-MMC and MAP30. The final obtained bioactive RIPs can be exploited for useful applications in clinical investigations and structural function studies, which can offer opportunities for future exploration of therapeutic agents.

## Materials and Methods

### Materials

*Momordica charantia L*. seeds were obtained from the Institute of Agricultural Science and Technique of Sichuan Province, China. Electrophoresis reagents were products of Sigma-Aldrich (St Louis, MO). Macro-Cap-SP, SP-Sepharose FF, Sephacryl S-100HR and ampholyte were purchased from GE Healthcare Bio-Sciences AB (Uppsala, SE). Dulbecco's Modified Eagle's Medium (DMEM) and fetal bovine serum (FBS) used in cell culture were from Gibco BRL (Grand Island, NE). The lung adenocarcinoma A549 cell line was obtained from American Type Culture Collection (ATCC CCL-185) (Manassas, VA). LMW Calibration Kit was supplied by SIBAS (Shanghai, China). pUC18 DNA used in detection of topological activity was obtained from TAKARA (Dalian, China). All other chemical reagents were standard commercial products of analytical grade.

### Purification of α-MMC and MAP30

The whole process was carried out at 4–6°C unless specifically stated. The sample pretreatment was according to previous methods with appropriate modifications [16], [17]. For typical preparation, four hundred grams of fresh and mature seeds of bitter melon were decorticated, pulverized to powder which was filtered at least three times through 0.5 mm sieve. Then the powder was extracted by adding 2.0 L of ice-cold 0.15 M NaCl solution containing 0.1% polyvinylpyrolidone with gentle stirring at 4°C overnight. The extract was filtrated through cheesecloth so as to remove wax and then centrifuged at 12,000 g for 10 min. The supernatant was adjusted pH to 3.6 by adding 2.0 M HAc-NaAc buffer. After removal of the precipitate by simple centrifugation, the supernatant was adjusted again to pH 6.3 with 3.0 M, pH 6.3 sodium phosphate buffer and this was the cell-free crude extract. The following purification step was 30%–60% ammonium sulphate saturation. The crude protein pellet from 60% ammonium sulphate saturation was dissolved in a minimal volume of Buffer A (pH 6.3, 50 mM sodium phosphate buffer containing 0.03%NaN_3_) and dialyzed against the same buffer in three changes (4 h for each). The protein solution was centrifuged to release any insoluble impurities and then applied to further purifications after dialysis. The samples from above steps were applied onto a SP-Sepharose FF column which was preequilibrated with buffer A at a flow rate of 2 mL/min. The column was eluted with approximately 5-fold column volume of buffer A to remove unabsorbed fraction until the OD_280 nm_ was lowered to baseline. Whereas, the bound proteins were eluted with 0.15 M NaCl in buffer A and the collected fraction was concentrated to 25–30 mg of protein per milliliter by Biomax 5 kDa membrane. Sephacryl S-100HR column (2.5 cm×120 cm) at flow rate of 1 mL/min was used as the second step. The column was eluted successively with 0.15 M NaCl in buffer A. The elution part detected by SDS-PAGE detected in 30 kDa was pooled and desalted by exchanging buffer A with Pellicon Biomax 5 kDa membrane. Finally, the collected fraction was subjected to Macro-Cap-SP column which was also previously equilibrated with buffer A followed by washing with the same buffer. A linear gradient from 0.01 M NaCl to 0.15 M NaCl in buffer A elution protocol was adopted and two protein peaks were collected. The final purified sample was 20–30 mg per milliliter using Biomax 5 kDa membrane for concentration. After filtrating with 0.22 µm membrane, the aliquots were stored at 4°C for subsequent research.

### Determination of protein content

Protein content was determined according to the method of Lowry [18] using bovine serum albumin (BSA) as standard or ultraviolet absorption at 280 nm for rough detection.

### Polyacrylamide Gel Electrophoresis

Acidic native PAGE was performed by the method of Niepmann [19]. Separation of the samples was implemented using discontinuous native polyacrylamide gels with 4.0% stacking gel and 12.0% running gel with pH 4.5 β-alanine-HAc buffer. The result was indicated by staining with Coomassie brilliant blue. SDS-PAGE was carried out according to the procedure of Laemmli [20] used a 12.0% resolving gel and a 5.0% stacking gel on a Mini Protean II apparatus (Bio-Rad). The gel stained for protein was applied with Coomassie brilliant blue and destained gel with 10% acetic acid and 25% methanol. LMW calibration kit was employed as standard for molecular weight evaluation.

### Isoelectric focusing electrophoresis

Determination of two purified RIPs was proposed on BIORAD Model II Mini IEF Cell in polyacrylamide gel with 5.0% and ampholyte with 2.0%. The voltage was increased with 100 v/15 min, 200 v/15 min and 450 v/60 min. Bands in the gels were stained by coomassie brilliant blue R-250 and confirmed pI was based on the distance against standard [21], [22].

### MALDI-TOF/TOF Mass Spectrometry and *N*-terminal sequence analysis

The molecular weights of α-MMC and MAP30 were detected on MALDI-TOF/TOF (Autoflex III,Bruker Corporation) [23]. Samples were prepared by mixing 0.5 µL of aliquot with 0.5 µL of the matrix solution (0.5 g/L CHCA in 50% of water/acetonitrile with 0.1% TFA acid). After dried by vacuum on the sample plate, data for 2 ns pulses of the 337 nm nitrogen lasers were averaged for each spectrum in a linear mode, and positive ion TOF detection was performed using an accelerating voltage of 20 kV. A proteolysis fragment of myoglobin was used as the external calibration. *N*-terminal sequences of the purified α-MMC and MAP30 were consigned to Chinese Academy of Agricultural Sciences. After HPLC analysis, the 5 amino acids of *N*-terminal were tested by Edman degradation method, respectively.

### Identification of glycoprotein and analysis of saccharide content

The glycoprotein nature of two RIPs was confirmed by the Periodate-Schiff's base (PAS) method previously described by Doerner [24]. The saccharide content was estimated with glucose as standard using anthrone-sulfuric acid method given by Graham [25].

### Topological inactivation activity of RIPs

To identify the topological inactivation activity of RIPs, the reaction was occurred with a final volume of 10 µL containing 200 ng of supercoiled pUC18 as a substrate. Supercoiled pUC18 and α-MMC or MAP30 were incubated at 37°C for 60 min. Added with suitable enzymatic conditions, the products were analyzed using electrophoresis in 1.0% agarose gel in Tris-phosphate buffer with Mg^2+^. The result was stained with ethidium bromide and destained in distilled water prior to photo documentation by using a short wavelength UV.

### Detection of Superoxide dismutase activity

It is recently reported that type I RIPs possessed superoxide dismutase (SOD) activity [4]. In order to define whether α-MMC and MAP30 own this, pyrogallol autoxidation method and NBT staining were used [26], [27]. Samples were assayed in a solution of air-equilibrated 0.6 mM pyrogallol, 100 mM pH 8.2 Tris-HCl buffer, and 1 mM EDTA, and the rate of pyrogallol autooxidation was measured with the Unico UV-2012 PC spectrophotometer. In order to determine the precision of the assay, three independent runs on the same sample were employed. One unit of enzyme activity was defined as the amount of the enzyme which represented 50% inhibition of the autoxidation rate of 0.6 mM pyrogallol at 25°C. Electrophoresis of the separated RIPs was performed using previous condition at 140 V until the bromphenol blue marker extended to the edge of the strip. SOD isoenzymes were visualized by NBT staining solution containing 2.45×10^−3^ M Nitroblue tetrazolium, 0.028 M Tetramethylethylenediamine, 2.8×10^−5^ M riboflavin and 0.05 M potassium phosphate in pH 7.8.

### Inhibitory effect of RIPs on cancer cell growth

Lung adenocarcinoma A549 cells were maintained in DMEM culture medium and supplemented with 10% heat-inactivated fetal bovine serum (FBS), 100 U/ml penicillin and 50 U/ml streptomycin in a 5% CO_2_ incubator at 37°C (Thermo Forma 3110, Waltham, USA). Quantitative 3-(4,5-dimethylthiozol-2-yl)-2,5-diphenyltetrazolium bromide (MTT) was applied to evaluate cell viability and proliferation. Cell concentration was adjusted to 1×10^4^ cells/ml after trypan blue staining and cell counting with a haemocytometer. The suspended cells were then plated onto 96-well plate at 100 µL/well. After 6 h of initial cell attachment, 20 µL of diluted stock solutions of α-MMC or MAP30 were added at final concentrations of 1, 2, 4 and 8 µM following incubation for 24, 48 and 72 h (4 replicas per concentration). Contrastingly, cells without adding RIPs were used as control. At the end of the treatment, 20 µL (5 mg/mL) of MTT was added to each well and the plates were incubated at 37°C for 4 h. As for each well, 100 µL of acidified isobutyl alcohol (0.04 M HCl in isopropanol) or dimethyl sulfoxide (DMSO) was added. The optical density (OD) was measured at a wavelength of 570 nm using an enzyme-linked immunoadsorbent assay (ELISA) plate reader (Model 680, Bio-RAD, Hercules, USA). The cell viability and proliferation were analyzed and compared with the controls. The percentage of inhibition was calculated by the following formula:
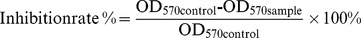



### Statistical analysis

The results were expressed as means of three independent measurements and were statistically evaluated using the standard deviation and *t* test methods. The difference was considered to be statistically significant when *P*<0.05.

## Results and Discussion

### Purification and identification of α-MMC and MAP30

**Figure 1:**
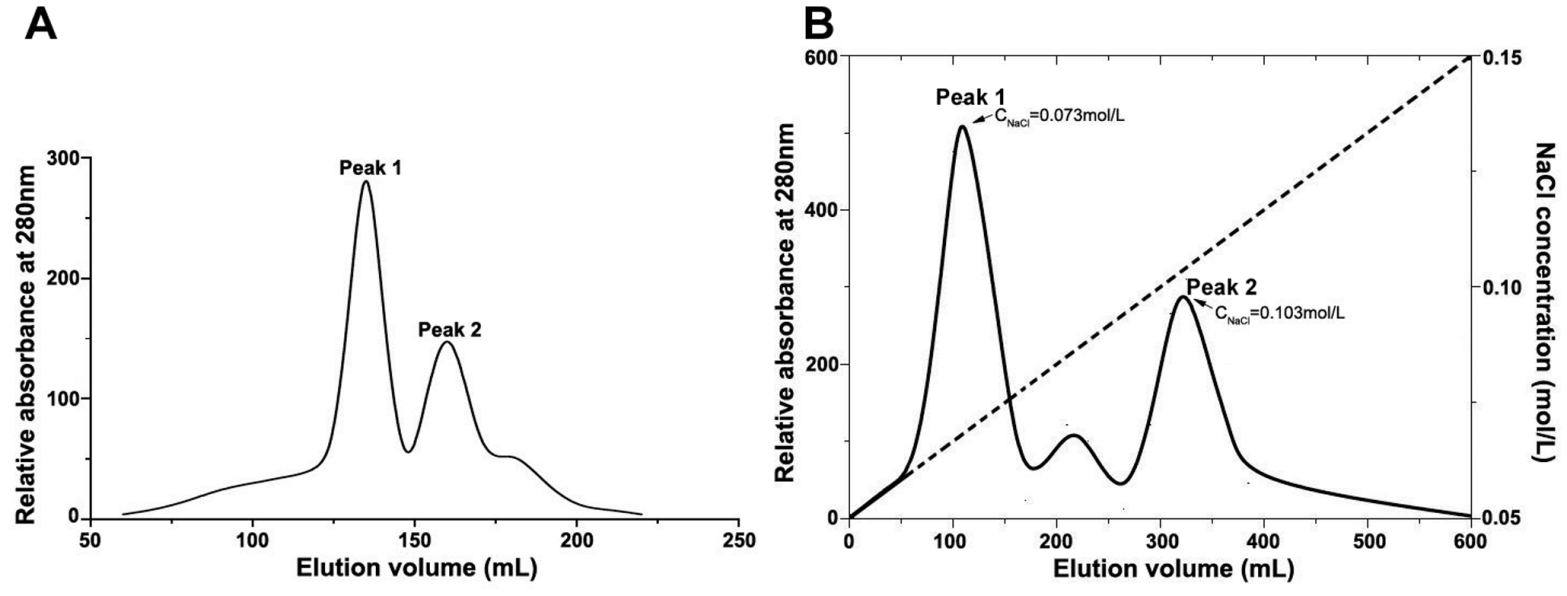
Elution profile of α-MMC and MAP30 on gel filtration and SP column. (A) Chromatography of fraction from SP-Sepharose FF on Sephacryl S-100HR column. After concentration, sample was added to the Sephacryl S-100HR chromatography (2.5 cm×120 cm) and eluted with a pH 6.5 10 mM sodium phosphate buffer containing 0.15 M NaCl at flow rate of 2 ml/min. Peak I was collected fraction. (B) Chromatography of fraction from Sephacryl S-100 column on Macro-Cap-SP Column. After desalting out and exchanging buffer of fraction from Sephacryl S-100, chromatography was loaded to Macro-Cap-SP column and eluted with 10 mM NaCl in pH 6.0 50 mM sodium phosphate. Finally a linear gradient from 50 mM NaCl to 150 mM NaCl in the same buffer at flow rate of 2 ml/min was used as eluent. Two fractions were collected and identified as α-MMC and MAP30, respectively.

Applying with this novel purification strategy, large amounts of impurities were removed by 30–60% ammonium sulfate precipitation and acidification steps. Both α-MMC and MAP30 were retained on SP-Sepharose FF column and eluted with 0.15 M NaCl in buffer A (pH 6.0, 10 mM sodium phosphate buffer, 0.03%NaN_3_). In gel filtration process, two major eluted peaks appeared, and the former was found to be protein with about 30 kDa (Fig. 1A). In order to separate α-MMC and MAP30, a specific Macro-Cap-SP chromatography was performed. From the result of Fig. 1B, it indicated that peak 1 was α-MMC which was eluted with NaCl from 0.059 M to 0.088 M and peak 2 was MAP30 from 0.098 M to 0.112 M. The whole purification process was summarized in Table 1. It concluded that 1162 mg of α-MMC and 535 mg of MAP30 can be obtained from 400 g starting material. The recoveries were 15.5% and 7.1%, respectively. Comparing with previous purification method, only 3.1 mg of α-MMC obtained from 2.5 g decorticated seeds. In other words, we can say that the efficiency of our purification strategy was about 2.34-fold for α-MMC [28] and there is no more report for large-scale preparation of MAP30. Additionally, this novel purification method can produce both α-MMC and MAP30 in a single process.

**Table 1:** Purification of α-MMC and MAP30 from 400 g of bitter melon seeds.

Steps	Total volume (mL)	Protein conc. (mg/mL)	Protein content (mg)	Recovery of Proteins (%)
Crude extracts	3023	-	-	-
pH 3.6 treatment	3000	2.5	7500	100
30–60% A.S. precipitation	208	16.3	3390	45.0
SP-Sepharose FF chromatography	200	15.6	3120	42.0
Sephacryl 100HR chromatography	158	16.0	2535	34.0
Macro-Cap-SP chromatography	α-MMC/MAP30			
	28/33	41.5/16.2	1162/535	15.5/7.1

**Note**: Data documented were the average value of 5 different preparations.

**Figure 2:**
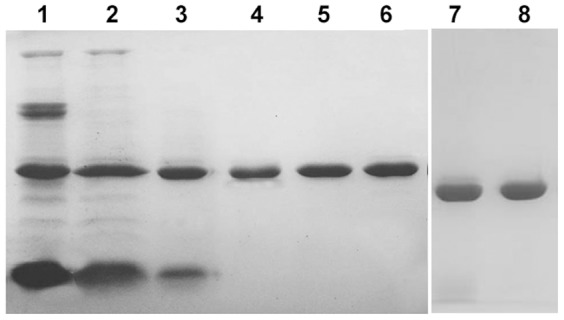
Analysis of extracts and eluents from different stages of purification by SDS–Polyacrylamide gel electrophoresis. Lane 1–6 was in the presence of 2-mercapoethanol. Lane 1 presented the crude extract after acidic treatment; Lane 2 presented the fractionation after 60% A.S. precipitation; Lane 3 presented the eluent from SP-Sepharose FF chromatography; Lane 4 presented the eluent from gel filtration chromatography; Lane 5 presented peak 1, 20 µg of α-MMC, from Macro-Cap-SP chromatography; Lane 6 presented peak 2, 20 µg of MAP30, from Macro-Cap-SP chromatography. Lane 7–8 was in the absence of 2-mercapoethanol. Lane 7 presented peak 1, 20 µg of α-MMC, from Macro-Cap-SP chromatography; Lane 8 presented peak 2, 20 µg of MAP30, from Macro-Cap-SP chromatography.

**Figure 3:**
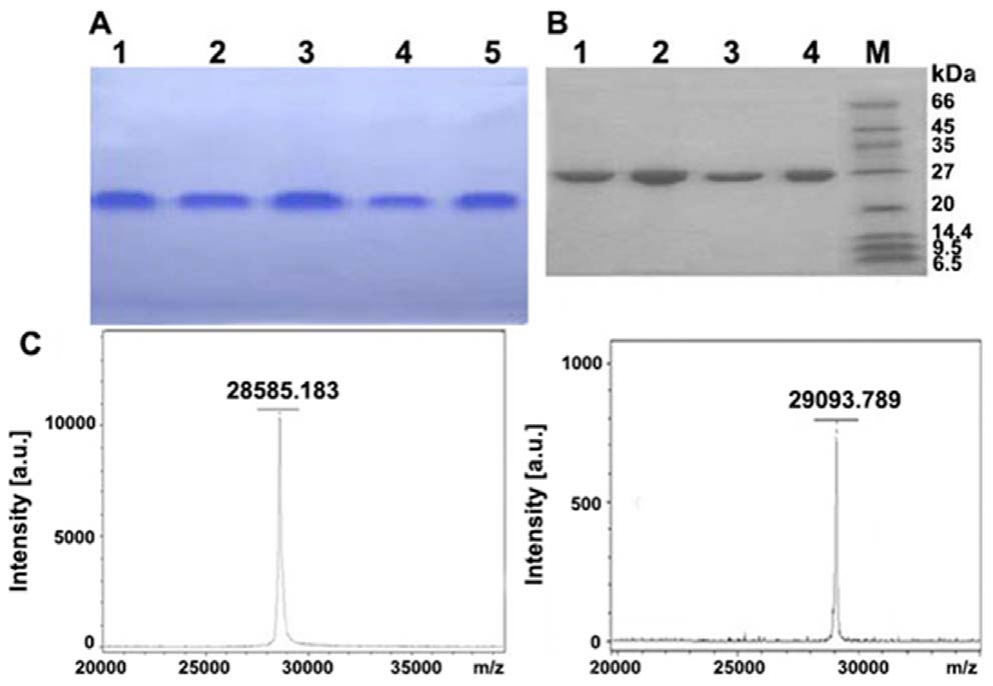
Size, homogeneity and subunit structure analysis of two purified proteins by electrophoresis and MALDI-TOF/TOF. (A) Homogeneity analysis of two purified proteins on acidic discontinuous native polyacrylamide gel electrophoresis on the basis of the charges of required proteins. Lane 1,2 presented 20 µg and 15 µg α-MMC; Lane 3,4,5 presented 20 µg,10 µg and 15 µg MAP30. (B) Analysis of the size, homogeneity and subunit structure of two proteins by SDS-PAGE in the presence and absence of 2-mercaptoethanol. Lane 1,2 indicated 10 µg and 20 µg α-MMC; Lane 3,4 indicated 10 µg and 20 µg MAP30; Lane 5 indicated LMW calibration kit (Phophorylase b 97 kDa, Albumin 66 kDa, Ovalbumin 45 kDa, Carbonic anhydrase 30 kDa, Trypsin inhibitor 20.1 kDa, α-Lactalbumin 14.4 kDa). (C) Molecular weight determination of α-MMC and MAP30. Left presented the analytic result for α-MMC on MALDI-TOF/TOF; Right presented the analytic result for MAP30 on MALDI-TOF/TOF.

**Figure 4:**
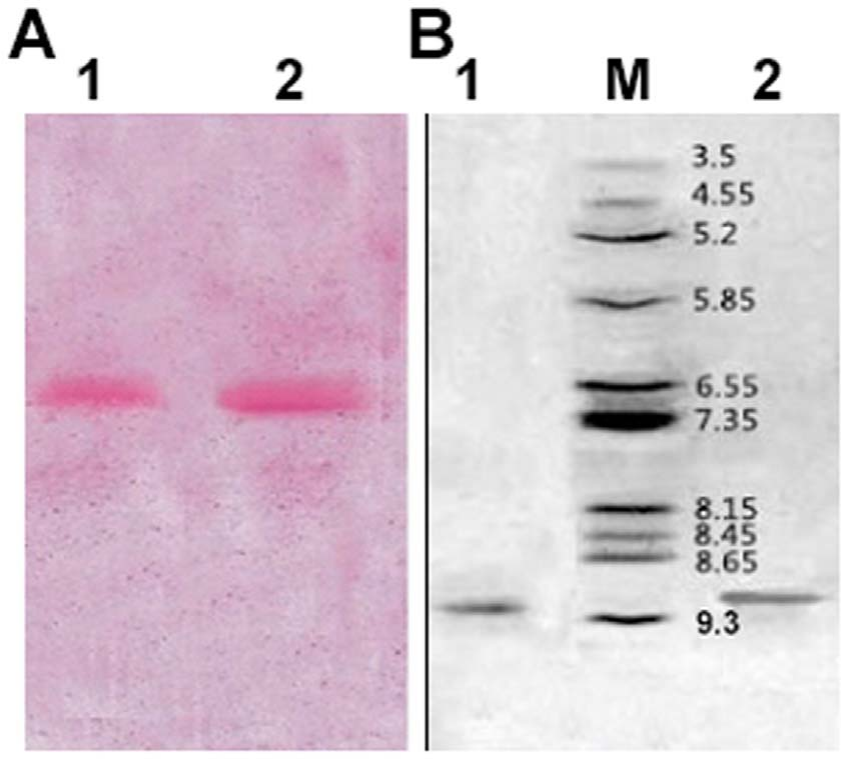
Identification of α-MMC and MAP30 as glycoprotein and pI analysis. (A) The identification of purified two proteins, α-MMC and MAP30, as glycoproteins by periodic acid-Schiff's staining on SDS-PAGE. Lane 1 and 2 presented the purified α-MMC and MAP30. (B) Determination of pI of these two proteins on IEF-Polyacrylamide gel electrophoresis. Lane1 and 2 presented the purified MAP30 and α-MMC, respectively. Lane M presented broad pI calibration kit with Amyloglucosidase 3.50, Soybean trypsin inhibitor 4.55, β-Lactoglobulin A 5.20, Carbonic anhydrase B bovine 5.85, Carbonic anhydrase B human 6.55, Myoglobin horse 7.35, Lentil lectin-acidic band 8.15, Lentil lectin-middle band 8.45, Lentil lectin-basic band 8.65, Trypsinogen 9.30.

The result of SDS-PAGE from different purification steps were shown in Fig 2. Further identification of homogeneity in charges was evaluated upon acidic PAGE (Fig. 3A) and molecular weight of 29–30 kDa on SDS-PAGE and MALDI-TOF/TOF (Fig. 3B&C). Staining gel with periodic acid-schiff has confirmed that these two RIPs were glycoproteins (Fig. 4A). It clearly showed that the bands of proteins were stained pink with periodic acid-schiff, which was the specific reaction of glycoproteins. Afterwards, the percentage of saccharides in both of them was estimated to be 1.25% and 1.1% with anthrone-sulfuric acid method. By using IEF analysis (Fig. 4B), pI of α-MMC and MAP30 were detected to be 9.02 and 9.12 on IEF-PAGE and the theoretical value was 9.13 and 9.08 by using the Compute pI/Mw program from ExPASy proteomics Server (http://web.expasy.org/compute_pi/). *N*-terminal sequence analysis indicated that α-MMC was N-Asp-Val-Ser-Phe-Arg and MAP30 was N-Asp-Val-Asn-Phe-Asp. The results were consistent with the database (http://www.ncbi.nlm.nih.gov/protein).

### Detection of Superoxide dismutase activity

**Figure 5:**
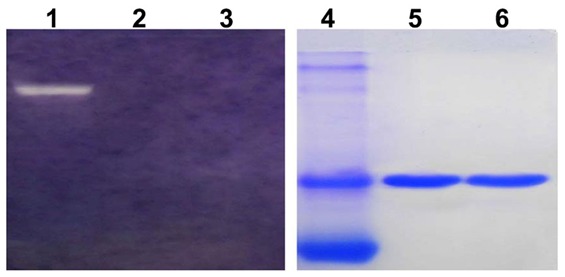
Detection of the SOD activity of crude extract, α-MMC and MAP30 on acidic PAGE. Lane 1∼3 stained by NBT; Lane 4∼6 stained by Coomassie brilliant blue R-250; Lane 1,4 presented crude extract of bitter melon seeds(50 µg of protein). Lane 2,5 presented α-MMC (20 µg). Lane 3,6 presented MAP30(20 µg).

In the reaction system of pyrogallol autoxidation method, the SOD activity of α-MMC and MAP30 was not detected in given 330 µg/mL of individual, while crude extract from bitter melon seeds was measured to be about 75 U/mL in the same condition, which was showed in Table 2. The SOD activity band cannot be observed on PAGE-NBT activity staining with 20 µg of α-MMC and MAP30. In the same condition, the obvious SOD activity band was found in 50 µg of the crude extract. The position of this band was not corresponded with the locations of α-MMC and MAP30 (Fig. 5). The above mentioned results indicated that α-MMC and MAP30 didn't possess SOD activity. However, some RIPs such as camphorin from *C. camphora* [29] and *C. moschata* RIP [30] exhibited SOD activity. Up to now, the reasons for this can not been fully explained. There appeared to be a need to look into deeply on these RIPs from different resources to fully understand their biological functions.

**Table 2:** Comparison of SOD activity in crude extract, α-MMC and MAP30.

Sample	Protein concentration (µg/mL)	Rate of pyrogallol autoxidation (ΔOD/min)	SOD activity (U/mL)
Crude extract	330	0.028±0.0009	75.25±2.2
α-MMC	330	0.056±0.001	0
MAP30	330	0.056±0.0004	0

**Note**: Each data represented the average of three independent experiments tested in quadruplicate.

### Effects of α-MMC and MAP30 on proliferation of lung adenocarcinoma A549 cells

**Figure 6:**
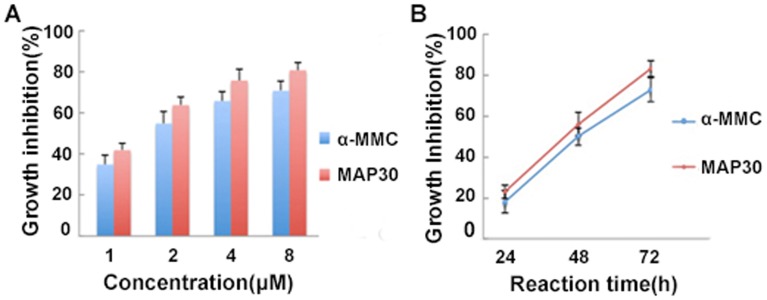
Inhibitory effects of α-MMC and MAP30 on the proliferation of A549 cells. (A) Cells were treated with increasing concentrations of 1, 2, 4 and 8 µM for 72 hours. (B) Cells were treated for various times of 24, 48 and 72 h at 8 µM. Note: Each data point represented the average of three independent experiments performed in quadruplicate. Error bars showed standard deviations (n = 3)

**Figure 7:**
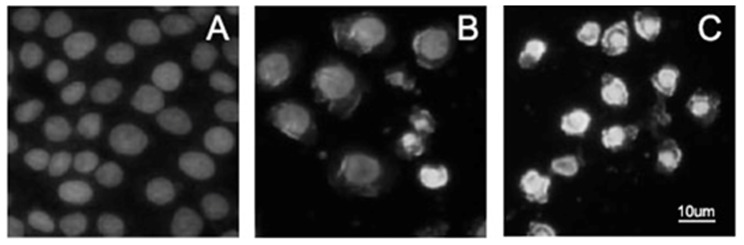
Morphological analysis of A549 cells treated by α-MMC and MAP30 after Hoechst33258 staining (×600). (A) Untreated cells as control. (B) Treated with α-MMC for 48 h. (C) Treated with MAP30 for 48 h.

To investigate the effect of both α-MMC and MAP30 on cell viability and proliferation, A549 cells were seeded on 96-well plates and were exposed to different concentrations of purified two RIPs for 72 hours (Fig. 6A) and for different times at 8 µM (Fig. 6B). Statistical analysis revealed that the concentrations of 8 µM can significantly reduce the proliferation of cells after 48 and 72 hours of incubation. The results indirectly demonstrated growth inhibition to A549 cells in a dose-and time-dependent manner. The result in Figure 6B also displayed that the growth inhibition ratios of cells was not prominent after treatment for 24 hours, but continuous incubation for 48 or 72 hours with the RIPs enhanced the cytotoxicity on cells. Additionally, α-MMC and MAP30 induced the apoptosis of A549 cells, which were detected using staining with Hoechst33258 under the inverted fluorescence microscopy (Leica, DMIL) (Fig. 7). The results showed that the normal A549 cells were extended and flattened (Fig. 7A), while the treated groups displayed nuclear enrichment, volume reduced and appearance of apoptotic bodies. In the cells treated with same concentration of RIPs, the photos have not expressed apoptotic morphology after 24 and 36 h. This revealed that A549 cells started to show apoptosis after 48 h treatment. Comparing with the proportion of the number of apoptotic cells, group of MAP30 (Fig. 7C) is more than the group of α-MMC (Fig. 7B).

### Topological inactivating activity

**Figure 8:**
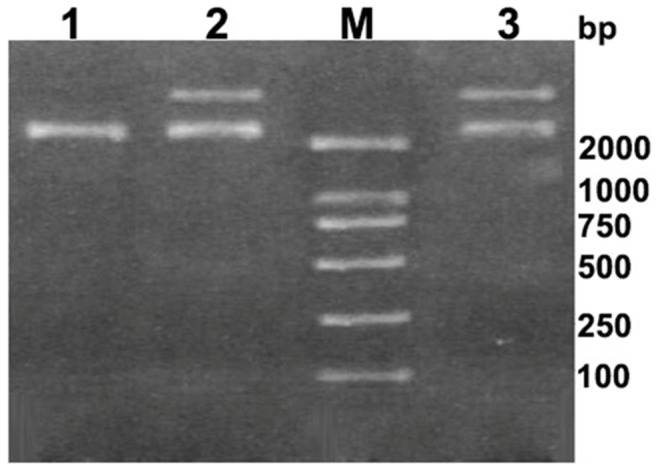
Topological inactivation activities. Lane 1 indicated pUC18 as a control; Lane 2 indicated the products of pUC18 DNA treated with α-MMC; Lane M indicated DNA ladder; Lane 3 indicated the products of pUC18 DNA treated with MAP30.

To demonstrate their topological inactivation activity, supercoiled DNA (pUC18) was exposed to α-MMC and MAP30, and pUC18 without RIPs treated was used as control. In suitable enzymatic digestion conditions, all of these proteins cleaved the supercoiled double-stranded DNA to produce nicked circular or linear DNA. As shown in Figure 8, all of them exhibited DNase-like activity.

## Conclusion

Studies described in this manuscript contributed a novel and simple purification strategy. The whole process contained only three purification steps and the identification analysis showed a high homogeneity and recovery for two RIPs. Additionally, α-MMC and MAP30 were confirmed to be glycoprotein. Anti-tumor activities of both RIPs were tested in lung adenocarcinoma A549 cell and the result showed an inhibitory effect. Finally, we also found that both two RIPs exhibited no superoxide dismutase (SOD) activity as other RIPs did. And this was firstly reported on α-MMC or MAP30 from *Momordica Charantia L*. Furthermore, these two RIPs also can convert supercoiled plasmid pUC18 DNA into relaxed forms which displayed a DNase-like activity. According to this novel purification method reported in this manuscript, affiliating with anti-tumor and anti-virus activities of α-MMC and MAP30, the results will facilitate subsequent researches on exploiting potential therapeutic agents.
